# Succinate-mediated metabolic alterations and signaling in RA: mechanisms and therapeutic implications

**DOI:** 10.3389/fimmu.2026.1842560

**Published:** 2026-07-15

**Authors:** Yi Fan, Naidi Wang, Chenxu Zhao, Zhaoqi Zhang

**Affiliations:** 1 Department of Rheumatology and Immunology, Beijing Key Laboratory of Non-invasive Diagnosis and Immunotherapy of Rheumatic Diseases, Peking University People’s Hospital, Beijing, China; 2 State Key Laboratory of Quantitative Synthetic Biology, Shenzhen Institute of Synthetic Biology, Shenzhen Institute of Advanced Technology, Chinese Academy of Sciences, Shenzhen, China

**Keywords:** HIF-1α, immunometabolism, RA, succinate, SUCNR1

## Abstract

Rheumatoid Arthritis (RA) is a chronic autoimmune disease characterized by persistent synovial inflammation. Recent studies have revealed that immune metabolites, particularly succinate, play a critical role in its pathogenesis. In this review, we systematically analyze the molecular mechanisms underlying succinate accumulation and elucidate how succinate drives inflammatory and immune dysregulation in RA. Hypoxia and inflammation microenvironment drive metabolic alterations, leading to succinate accumulation via three primary mechanisms: 1) succinate retention caused by impaired mitochondrial oxidation, 2) enhanced succinate synthesis through alternative biosynthetic pathways, and 3) increased succinate export into the extracellular space. Accumulated succinate exerts its pathogenic effects through three distinct pathways: (i) binding to its receptor Succinate Receptor 1 (SUCNR1) as an extracellular signaling molecule; (ii) stabilizing Hypoxia-Inducible Factor-1α (HIF-1α) in the intracellular space; and (iii) acting as a key epigenetic modulator. These insights into succinate-mediated pathogenesis provide a rationale for the development of targeted RA therapies, including strategies aimed at SUCNR1, Succinate Dehydrogenase (SDH), and the combination of these metabolic interventions with existing anti-inflammatory treatments.

## 1 Introduction

RA is a chronic and progressive autoimmune disease characterized by the infiltration of immune cells, the release of inflammatory effectors, and the pathological activation of synovial fibroblasts. These processes drive the irreversible degradation of articular cartilage and bone, which gradually leads various comorbidities (1). Currently, persistent inflammation and joint destruction in RA remain critical clinical challenges. The incidence of RA in China is between 28 and 40 cases per 10,000 population. It affects more than 5 million individuals overall, with a disability rate of up to 77.6% (2–4).

Metabolic pathways have emerged as central orchestrators of immune and stromal cell behavior in the inflamed joints of RA. This extensive metabolic reprogramming is primarily driven by the chronic hypoxia that characterizes the synovial microenvironment which underlies the metabolic shift toward a hypermetabolic phenotype (5–8). This shift occurs primarily to meet the biosynthetic demands of proliferation, but it also contributes to the abnormal accumulation of key metabolic intermediates.

Metabolomic analysis results provide direct evidence for this: the accumulation of metabolites can act as important signaling molecules and play an active role in inflammatory regulation and immune responses, thereby exacerbating chronic inflammatory disorders (8). For example, lactate accumulated in the synovial fluid of RA has been shown to induce T cells to produce interleukin-17A, thus amplifying inflammatory signals (9). Notably, the levels of Tricarboxylic Acid Cycle (TCA) intermediates, especially succinate, are generally elevated in the synovial fluid of RA patients (10, 11). This suggests that succinate is beyond its classical role as a metabolic intermediate and may participate in the disease process as a key “immune metabolite”.

Meanwhile, *in vitro* studies have found that succinate can promote the stabilization and activation of HIF-1α, and induce macrophages and other cells to secrete the potent pro-inflammatory cytokines (12, 13). These cytokines further activate additional immune cells such as macrophages and synovial fibroblasts, and promote osteoclast differentiation ultimately forming a vicious cycle that drives synovial inflammation, exacerbates cartilage destruction, and aggravates bone erosion (14, 15).

In conclusion, metabolic dysfunction represented by succinate is closely associated with progression, and severity of RA. In this review, we will first elaborate on the mechanisms by which the synovial microenvironment in RA drives metabolic alterations in key cells, leading to abnormal succinate accumulation. Then, we will systematically analyze the molecular mechanisms underlying this accumulation and elucidate how succinate may contribute to inflammatory and immune dysregulation. Finally, we will focus on succinate metabolism as an emerging therapeutic target in RA.

## 2 Succinate-associated metabolic alterations in immune cells

Succinate is not merely a consequence of metabolic shifts in immune cells including macrophages, fibroblast-like synoviocytes and T cells but also to actively regulate their inflammatory function by changing metabolic flux such as glycolysis and glutaminolysis and metabolite levels, thereby establishing a positive feedback loop. The metabolic characteristics and functional outputs of key immune cells are summarized as follows (**Table 1**):

**Table 1:** Metabolic and functional features of major immune cells in the RA synovial microenvironment.

Effector cell	Metabolic features	Functional features	Reference
FLS	Enhanced glycolysis	Coupled with glutamine metabolism to sustain the invasive phenotype of FLS.	(16–18)
	Increased glutaminolysis	Prominent under metabolic stress, supporting the aggressive phenotype of FLS and indirectly maintaining inflammation.	(16–18)
M1 macrophages	Enhanced glycolysis	M1 macrophages rely on high glycolytic flux.	(19, 20)
	Reduced oxidative phosphorylation	Impaired oxidative phosphorylation leads to ROS production and metabolite accumulation ,for example succinate, which directly activate the NLRP3 inflammasome and HIF-1α signaling axis, thereby exacerbating inflammation.	(21–23)
CD8^+^ T cells	Increased aerobic glycolysis and glutaminolysis	Enhanced glycolysis confers proliferative capacity under hypoxia and low-glucose conditions, closely associated with pro-inflammatory and cytotoxic phenotypes. LDHA inhibition may attenuate their function	(24)
	Enhanced fatty acid oxidation	Metabolic reprogramming toward lipid utilization provides energy and biosynthetic precursors necessary for sustained activation, expansion, and cytotoxicity of effector CD8^+^ T cells.	(25)
CD4^+^ T cells	Glycolytic flux redirected to PPP; reduced oxidative phosphorylation	Glucose metabolism is actively redirected toward the PPP, enhancing antioxidant capacity but disrupting ROS signaling required for T-cell activation, ultimately driving abnormal differentiation toward pro-inflammatory Th1/Th17 cells.	(26-28)
	Increased glutaminolysis	Under metabolic stress, elevated glutamine metabolism may support survival and function in the hypoxic and low-glucose synovial microenvironment	(16–18)

### 2.1 Succinate-associated metabolic alterations in macrophage

In macrophages, hypoxia and the accumulation of Reactive Oxygen Species (ROS) stabilize HIF-1α synergistically, driving a metabolic shift from Oxidative Phosphorylation (OXPHOS) to glycolysis during the RA progression. Therefore, macrophages in RA exhibit a pro-inflammatory phenotype due to this shift (19– 22). HIF-1α upregulates the expression of glycolytic key enzymes, such as Hexokinase 2 (HK2) and Lactate Dehydrogenase A (LDHA), leading to enhanced lactate production (29). *In vitro* studies have shown accumulated lactate can activate the Nuclear Factor Kappa B (NF-κB) pathway in macrophages. Additionally, Mitochondrial dysfunction promotes NOD-like receptor family, pyrin domain containing 3 (NLRP3) inflammasome activation. Together, these processes result in the excessive production of cytokines such as IL-1β and Tumor Necrosis Factor-α (TNF-α) (23). However, the regulatory role of succinate in macrophage polarization toward pro-inflammatory or anti-inflammatory phenotypes is complex. For example, Littlewood-Evans et al. reported that in the AIA mouse model the acute accumulation of extracellular succinate after an inflammatory stimulus activates SUCNR1 in macrophages, thereby amplifying their pro−inflammatory response. In human RA synovial fluid, succinate levels correlated with IL-1β-inducing activity, and this effect was blocked by a GPR91 antagonist in a human myeloid cell line (U937). These together indicate that the succinate–SUCNR1 signaling axis may form a feed-forward loop that exacerbates inflammation (12). In contrast, distinct work by Mette Trauelsen and colleagues revealed an anti-inflammatory dimension of this axis. In their study, treating anti−inflammatory (M2−like) macrophages with 500 µM succinate for 24 hours hyperpolarized their M2−like phenotype via SUCNR1−mediated Gq signaling (30). These collective findings underscore that the succinate–SUCNR1 axis can orchestrate divergent immune outcomes ranging from pro−inflammatory amplification to anti−inflammatory reinforcement, depending on the macrophage polarization state which means the balance between pro-inflammatory M1-like and anti-inflammatory M2-like phenotypes, succinate concentration and exposure dynamics, the dominant signaling pathway engaged and the surrounding metabolic microenvironment.

### 2.2 Succinate-associated metabolic alterations in fibroblast-like synoviocytes

FLS are central effect cells in joint destruction. Within the RA synovium, these fibroblasts undergo profound metabolic alteration, a process characterized by upregulated Glucose Transporter 1 (GLUT1) expression and enhanced activity of key glycolytic enzymes such as HK2 and 6-phosphofructo-2-kinase/fructose-2,6-biphosphatase 3 (PFKFB3) that drive a distinct increase in glycolytic flux (16, 31–33). While this increase in glycolysis may be associated with succinate accumulation in other cell types (21), direct evidence for this theory in FLS remains insufficient. Notably, FLS exhibit metabolic flexibility, which enables them to integrate heightened glycolysis with glutaminolysis to maintain their invasive phenotype (16–18). It is plausible that this metabolic shift, particularly the enhanced glutaminolysis, could indirectly contribute to larger succinate accumulation, though it remains a hypothesis. More importantly, FLS serve as central responders to succinate signaling. The mitochondrial dysfunction and ROS production during their metabolic alterations may cooperate with succinate accumulation to activate critical signaling axes, such as the HIF-1α pathway (14, 34). This activation subsequently upregulates the expression of matrix metalloproteinases (MMPs) and Vascular Endothelial Growth Factor (VEGF), which together drive cartilage destruction and pannus formation, processes that are core in structural joint damage in RA (34). Furthermore, activated FLS are not merely passive responders. The diverse factors they secrete, such as the supernatant following TNF-α stimulation, can exert influence on macrophages, promoting a pro-inflammatory metabolic phenotype in these cells (22). This interaction establishes a positive feedback loop between cells, which perpetuates the amplification of the inflammatory microenvironment and may further stimulate the production and release of succinate.

### 2.3 Succinate-associated metabolic alterations in T cells

T cells in RA patients undergo metabolic alterations, characterized by a reduced reliance on oxidative metabolism. These changes are closely associated with enhanced pathological activation, proliferation, differentiation, and migratory capacity (24). Metabolic alterations vary across T cell subsets, especially in CD4^+^ and CD8^+^ T cells. Succinate has been shown to affect adaptive immune cells in addition to its role in innate immunity and FLS biology (35–39). It links metabolite production to immune responses. Furthermore, studies have demonstrated that succinate has a regulatory effect on T cell function and differentiation in diverse disease models including tumor immunology, inflammatory bowel disease, and renal fibrosis.

Unfortunately, direct evidence for succinate’s effect on T cells in RA is currently limited. Future studies may focus on the role of succinate in T cell metabolic alterations in RA patients, where succinate could represent a novel metabolic target to curb pathological T cell activation.

#### 2.3.1 Metabolic characteristics of CD4^+^ T Cells in RA

CD4^+^ T cells in RA patients exhibit a “hypometabolic” state: their oxygen consumption and lactate production are both less than those of healthy controls, resulting in reduced Adenosine Triphosphate (ATP) production and less intracellular ATP (26–28). This is because CD4^+^ T cells in RA patients divert from glucose toward the pentose phosphate pathway (PPP), which is achieved by the alteration of the enzyme activity of two key rate-limiting enzymes. The expression of glycolysis-promoting PFKFB3 is reduced in RA CD4^+^ T cells (26), while the rate-limiting enzyme of the PPP, Glucose-6-Phosphate Dehydrogenase (G6PD), is overexpressed (28). This shift results in elevated Nicotinamide Adenine Dinucleotide Phosphate (NADPH) production and glutathione levels, enhancing their anti-oxidant capacity. However, it impairs the intracellular ROS signaling required by T Cell Receptor (TCR) activation. Disruption of this signaling pathway ultimately drives naive T cells toward aberrant differentiation into pro−inflammatory T Helper 1 cell (Th1) and T Helper Cell 17 (Th17) subsets, thereby exacerbating disease progression (26, 28).

Direct evidence for succinate’s effects on CD4^+^ T cells in RA is currently lacking. However, studies in other diseases suggest several mechanisms that may be relevant.

In T cells lacking the Succinate Dehydrogenase Subunit B (SDHB), succinate accumulates, raising the succinate/α-Ketoglutarate (α-KG) ratio. This inhibits histone and DNA demethylase activity, thereby remodeling chromatin accessibility via a HIF-1α independent pathway (35). In fact, succinate acts as an epigenetic modulator within T cells, influencing their differentiation. Succinate accumulation may specifically upregulate key transcription factors such as PR Domain Containing 1 (Prdm1), directly driving T−cell differentiation toward the Th1 and Th17 lineages and promoting pro−inflammatory gene expression. Emerging research in renal fibrosis indicates that succinate may act within CD4^+^ T cells to alter their gene expression profile, driving the production of C-C Motif Chemokine Ligand 1 (CCL1) and thereby promoting fibroblast activation and renal fibrosis (36). Moreover, in inflammatory bowel disease (IBD) models, recent research has revealed that elevated extracellular succinate can severely impair Regulatory T Cell (Treg) cell function through a SUCNR1-independent axis. Succinate enters the cell and downregulates Oxoglutarate Dehydrogenase Complex (OGDHC), reducing succinyl-CoA, which impairs Forkhead Box P3 (FOXP3) succinylation, promoting STUB1-mediated K48-ubiquitination and proteasomal degradation (37).

Especially, a study in RA shows that Intraperitoneal succinate administration exacerbated arthritis in mice, whereas this effect was reversed in SUCNR1-deficient mice (40). Succinate can indirectly influence CD4^+^ T−cell polarization by modulating dendritic cells (DCs): exogenous succinate can act on the DC membrane receptor SUCNR1, promoting Th17 cell expansion by enhancing DC infiltration into draining lymph nodes (40, 41).

Collectively, these findings from different models suggest that succinate signaling may potentially contribute to the functional regulation of CD4^+^ T cells in RA, a role that merits further investigation.

#### 2.3.2 Effects of succinate on CD8^+^ T cells

In contrast to CD4^+^ T cells, reports on the effects of succinate on CD8^+^ T cells remain limited. CD8^+^ T cells from RA patients present a proinflammatory phenotype and have an activated energetic metabolism (42). Studies indicate that CD8^+^ T cells from RA patients undergo a metabolic shift toward aerobic glycolysis and glutaminolysis. All CD8^+^ T−cell subsets overexpress enzymes associated with Warburg effect, including LDHA. This metabolic alterations enables the cells to proliferate under hypoxic and low glucose conditions and is closely linked to their proinflammatory phenotype. Targeting and inhibiting LDHA effectively attenuates their inflammation and reduces tissue damage (24). The impact of succinate on CD8^+^ T cells in RA also remains unclear.

In RA, CD8^+^ T cells produce various effector molecules such as granzyme B, perforin and interferon−γ, which promote local inflammatory responses and aggravate the disease process (43–49). In tumors, lactate can induce a metabolic shift from PC to Pyruvate Dehydrogenase (PDH). Under these conditions, CD8^+^ T cells exhibit increased SDH activity, which consumes succinate through TCA cycle rather than exporting it to activate SUCNR1 signaling. Succinate, which is generated indirectly by Pyruvate Carboxylase (PC) and actively exported can rapidly upregulate the expression of granzyme B, perforin, and Interferon-γ via SUCNR1 signaling. Elevated levels of inflammatory molecules in CD8^+^ T cells, induced by succinate/SUCNR1 signaling, may promote the progression of RA.

In summary, current research suggests that although succinate may influence CD8^+^ T cells through multiple mechanisms, its specific role in CD8^+^ T cells in RA remains to be fully elucidated, particularly its connection to metabolic alterations. Future studies need to clarify the role of succinate metabolism in RA CD8^+^ T-cell alterations which may represent a novel metabolic target for intervening in aberrant T-cell activation.

## 3 Succinate levels are increased in patients with RA

The elevated succinate levels in the synovial fluid of RA patients (10) serve as the foundation for its role in inflammatory signaling. This accumulation is caused by cellular metabolic alterations induced by the inflammatory and hypoxic synovial microenvironment, and is mainly accomplished through three mechanisms. The mechanisms reported in RA include impaired mitochondrial oxidation leading to succinate retention, and active export of succinate into the extracellular space. Meanwhile, activation of alternative biosynthetic pathways that increase succinate production has also been reported, primarily in other inflammatory models like ischemia and infection models1 (10).

### 3.1 Succinate retention caused by impaired mitochondrial oxidation

Succinate is a key intermediate in the TCA and is largely generated from α-KG within the mitochondrial matrix. Under physiological conditions, it is promptly oxidized to fumarate by SDH, a process that couples to the electron transport chain for ATP generation (50). However, under inflammatory or hypoxic conditions, the activity of SDH can be impaired or even reversed, which reduces fumarate back to succinate, establishing a reversible “overflow” pathway (51–54). Direct evidence for SDH reversal in RA has been obtained in synovial fibroblasts under hypoxic and TGF-β1 stimulation (14).

### 3.2 Release of succinate from cells into the extracellular space

Accumulated succinate is not retained within the cell. Activated macrophages, among others, actively release it into the extracellular space (12). Elevated concentrations of extracellular succinate have been detected in the synovial fluid of RA patients. Evidence indicates that endogenous Toll-like receptor (TLR) ligands within RA synovial fluid can activate macrophages, which both drives glycolysis to increase endogenous succinate production and promotes its release. The released succinate can then act in an autocrine or paracrine manner by binding to cells which express its specific receptor, SUCNR1, as a result of enhancing IL−1β production (12). Notably, both LPS and IL-1β can upregulate the expression of SUCNR1, thus establishing a positive feedback loop that sustains and exacerbates inflammatory signaling (12).

### 3.3 Enhanced succinate production via alternative pathways

In addition to mitochondrial metabolic imbalance, the *de novo* synthesis of succinate is markedly increased in activated immune cells. Here, “glutamine-dependent anaplerosis” serves as a key alternative pathway. Under stimuli such as lipopolysaccharide (LPS), macrophages increase succinate production by enhancing glutamine catabolism through an undescribed mechanism (13, 55, 56). Furthermore, specific studies have further delineated the relevant pathway: in macrophages infected with mycobacteria, TNF enhances glutamine transport and breakdown via the receptor-interacting protein kinase 3 (RIP3)/phosphoglycerate mutase Family member 5 (PGAM5) signaling axis, leading to succinate accumulation (55). Notably, TNF−α is also a key inflammatory cytokine highly expressed in RA synovial fluid, whether this pathway plays a role in RA remains unverified. Additionally, under anaerobic conditions, the Gamma-Aminobutyric Acid (GABA) shunt pathway may also contribute to succinate production (13, 57). Furthermore, during ischemia, activities like the malate−aspartate shuttle or the purine−nucleotide shuttle can raise intramitochondrial fumarate levels (56, 58, 59). SDH can operate in the reverse direction, catalyzing the reduction of fumarate to succinate (56). This forms a reversible overflow pathway. These metabolic adaptations drive a sharp rise in endogenous succinate levels (13). Collectively, these mechanisms may represent an important cause of succinate accumulation in RA. However, while these alternative pathways are well established in models of ischemia, infection, their relative contribution to succinate accumulation specifically in the RA synovial microenvironment has yet to be directly addressed.

## 4 Regulatory pathways of succinate in immune homeostasis in RA patients

In RA, succinate is not merely an accumulated metabolic intermediate, but rather a central mediator to inflammation, tissue destruction, and immune imbalance through both intracellular and extracellular signaling pathways.

### 4.1 The SUCNR1 signaling axis: an extracellular pro-inflammatory positive feedback loop

Extracellular succinate, functioning as a danger signal molecule, directly mediates the activation of innate immunity cells by binding to its specific G-protein coupled receptor, SUCNR1, also known as G Protein-Coupled Receptor 91 (GPR91). SUCNR1 is highly expressed on the surface of macrophages and dendritic cells, whereas this receptor is rarely observed on the surface of T and B lymphocytes (41). SUCNR1 is an inhibitory G protein-coupled receptor (GPCR), specifically a subunit-associated GPCR (41, 60).

In the RA synovium, endogenous TLR ligands first activate macrophages, leading to enhanced glycolysis, increased endogenous succinate production, and the concurrent release of succinate into the extracellular environment (12). This succinate then acts in an autocrine or paracrine manner by binding to SUCNR1, which is expressed on the surface of macrophages and other cells, thereby significantly enhancing the production of interleukin-1β (IL-1β) (12). This pathway establishes a self-amplifying positive feedback loop. On one hand, upstream inflammatory signals (e.g., LPS) and the downstream product IL-1β can both upregulate SUCNR1 expression, sensitizing cells to succinate; On the other hand, sustained SUCNR1 activation further perpetuates the inflammatory state. Genetic or pharmacological blockade of this pathway (e.g., via SUCNR1 deletion or antagonist use) markedly reduces IL-1β secretion. Moreover, clinical studies show that succinate levels in RA synovial fibroblasts correlate positively with both IL-1β concentration and joint swelling scores, confirming the critical role of this axis in sustaining inflammation (12).

Extracellular succinate induces tumor angiogenesis via SUCNR-1-mediated activation of Extracellular Signal-Regulated Kinase 1/2 (ERK1/2) and Signal Transducer and Activator of Transcription 3 (STAT3), thereby upregulating VEGF expression (61).

### 4.2 HIF-α axis-mediated intracellular signaling

Intracellular succinate stabilizes HIF-α through two main mechanisms.

First, succinate inhibits the activity of Prolyl Hydroxylase (PHD), thereby preventing HIF-1α from undergoing ubiquitin-mediated degradation (62, 63). In some situations, this promotes the stabilization of HIF-1α even in the presence of oxygen, a phenomenon known as pseudohypoxia (16, 63).

Second, succinate stabilizes HIF-1α by amplifying ROS signaling through two distinct mechanisms. Succinate drives mitochondrial ROS production by fueling reverse electron transport at complex I following its oxidation by SDH (64). Besides, succinate impairs cellular antioxidant defenses. Its accumulation depletes NADPH and glutathione, which reduce the capacity to scavenge ROS resulting in ROS-driven signaling (65). In the hypoxic microenvironment of RA synovium, hypoxia-induced ROS accumulation also enhances HIF-1α synthesis via the GRK2 axis (66). Together, these mechanisms represent the central intracellular pathway through which succinate drives inflammation. The stabilized HIF-1α translocates to the nucleus, where it acts as a transcription factor to exert multiple pro−inflammatory effects:

1. Activation of the NLRP3 inflammasome: in FLS, the succinate–HIF−1α axis acts as a key upstream signal for NLRP3 inflammasome activation. Knockdown of HIF−1α leads to reduced NLRP3 expression and decreased maturation of IL−1β (14). Substantial histological evidence confirms that HIF-1α expression increases in parallel with the degree of inflammation in both the lining and sublining layers of the RA synovium (33, 67).
2. HIF−1α drives both an invasive phenotype and angiogenesis: it directly upregulates VEGF, promoting pathological synovial neovascularization (pannus formation) that enables inflammatory cell infiltration and pannus expansion. Concurrently, HIF−1α synergizes with signals such as NF−κB to upregulate MMPs, thereby enhancing the invasive capacity of FLS and directly contributing to cartilage and bone erosion (68, 69).
3. During pathological tissue remodeling, hypoxia or Transforming Growth Factor-β1 (TGF-β1) may induce functional modulation of SDH, exacerbating succinate accumulation in synovial fibroblasts. Succinate then promotes IL-1β secretion via the previously mentioned HIF-1α/NLRP3 axis, while IL-1β in turn upregulates profibrotic factors such as TGF-β1, thereby establishing a self-perpetuating cycle from inflammation to fibrosis (14).

In the RA synovium, HIF-1α and NF-κB drive chronic inflammation through bidirectional crosstalk (70). Under hypoxia, phosphorylation of the IκB kinase (IKK) complex initiates the canonical NF-κB pathway, leading to Inhibitor of nuclear factor kappa B alpha (IκBα) degradation and release of p65/p50 for nuclear translocation (71, 72). Nuclear NF-κB can directly bind to the HIF-1α promoter to upregulate its expression (71, 73). HIF-1α then binds to the hypoxia response element (HRE) in the NLRP3 gene promoter, triggering inflammation, a process named “NF-κB-HIF-1α-NLRP3” axis, which has been validated in RA synovial fibroblasts (66). In addition, NF-κB can also directly upregulate NLRP3 expression to provide inflammasome priming signals (74).

Hypoxia-induced ROS accumulation exerts dual amplifying effects in FLS of RA patients: on one hand, it promotes IκBα degradation to sustain NF-κB activation in synovial fibroblasts (71); on the other hand, it enhances HIF-1α synthesis via the G protein-coupled receptor kinase2 (GRK2) axis (66). In summary, ROS amplification, IKK-dependent NF-κB activation, and HIF-1α-mediated NLRP3 priming together constitute the core mechanism of metabolic reprogramming driving synovial inflammation in RA.

### 4.3 Succinate acts as a key epigenetic modulator

Succinate is a product of 2-Oxoglutarate-Dependent Dioxygenase (2-OGDD) enzyme reactions. By acting as a competitive inhibitor of these enzymes, accumulated succinate has a profound impact on histone and DNA demethylation (50). TET (ten-eleven translocation) DNA demethylases can suppress osteoclast activation and bone destruction through epigenetic modifications thereby influencing RA (75). Meanwhile, succinate has been reported to affect TET through epigenetic regulation in tumors. Succinate inhibits TET DNA demethylases and Jumonji C (JmjC) domain-containing histone lysine demethylases, thereby promoting DNA hypermethylation and histone hypermethylation. In T cells, the impact of succinate on epigenetic regulation has been directly demonstrated (76). T cells Lacking succinate dehydrogenase subunit B (SDHB), succinate accumulates and increases the succinate/α−ketoglutarate ratio. This inhibits histone and DNA demethylase activity, leading to increased H3K4me3 at the Prdm1 locus and promoting differentiation toward Th1 and Th17 lineages (35). Similar epigenetic mechanisms may operate in RA synovial cells, but direct evidence remains to be established.

**Figure 1:**
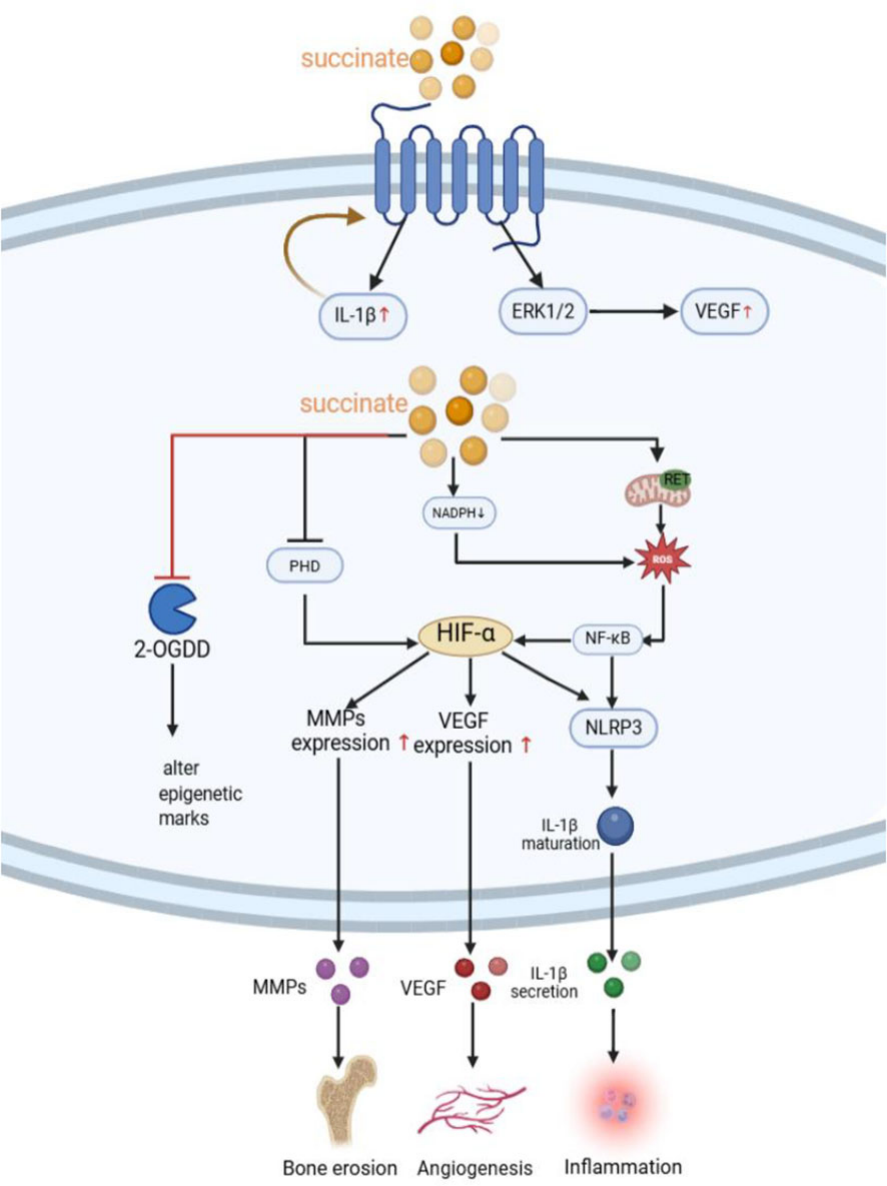
Succinate Promotes Inflammation, Angiogenesis, and Bone Erosion. Succinate drives RA pathology via three interconnected axes. Extracellular SUCNR1 Signaling: Activates ERK1/2 via GPR91 to induce IL-1β and upregulate VEGF, promoting inflammation and angiogenesis; Intracellular HIF-1a Stabilization: Intracellular Axis: Succinate inhibits PHD and induces mitochondrial RET or consumes NADPH to generate ROS. ROS then activate NF-KB, which both stabilizes HIF-1a. HIF-1a and NF-KB further activates NLRP3. Together, these events promote IL-1β maturation, angiogenesis, and bone erosion. Metabolic-Epigenetic Amplification: Inhibits 2-OGDD enzymes, altering epigenetic marks to sustain pro-inflammatory gene expression. These axes form a self-amplifying network driving chronic inflammation, pannus formation, and joint destruction.

In summary, elevated cytosolic succinate levels are likely to alter multiple epigenetic marks, thereby exerting long-term effects on gene expression (**Figure 1**) (76).

## 5 Therapeutic strategies targeting succinate pathways

Therefore, the dysregulation of metabolic reprogramming and succinate signaling represents an important therapeutic target in RA.

### 5.1 SUCNR1 antagonists

Future therapeutic strategies for RA may involve inhibiting extracellular succinate signaling. In animal models, inhibitors of the succinate receptor SUCNR1 have been shown to reduce dendritic cell migration, attenuate Th17 cell expansion, and alleviate arthritis-associated symptoms (40). However, the complexity of the succinate signaling axis presents a significant challenge (30, 77). Broad inhibition of succinate receptors risks disrupting systemic metabolic homeostasis. More importantly, the precise role of succinate in human RA patients remains incompletely understood. Despite these challenges, targeting SUCNR1 with specific inhibitors represents a promising novel therapeutic avenue for RA.

### 5.2 Metabolic regulation

T cells with SDHB deficiency exhibit an elevated succinate/α-KG ratio, which epigenetically drives the differentiation toward Th1 and Th17 subsets (35). Modulating SDH activity may therefore correct immune imbalance. The downstream target of HIF-1α plays a central role in this process. Its stabilization promotes the invasiveness of FLS and the expansion of T helper cells (68, 78, 79). In theory, inhibiting HIF-1α or NLRP3 can mitigate multi-pathway-mediated tissue damage. Studies have demonstrated that cinnamaldehyde (CA) may serve as a potential therapeutic compound. CA reduces intracellular succinate accumulation and downregulates HIF-1α expression, leading to decreased IL-1β production and attenuated progression of RA (80). However, this approach requires addressing challenges in target specificity and avoiding potential interference with physiological hypoxic responses.

### 5.3 Combination therapy approaches

Combining such metabolic interventions with existing therapies like anti-TNF-α is rational and may synergistically inhibit the inflammatory network. Insights from tumor immunology suggest that modulating T cell metabolic adaptability via circuit concerning succinate could be a promising adjunctive strategy (38). These approaches may also be combined with immunomodulatory therapies to restore immune homeostasis.

## Glossary

α-KG: α-Ketoglutarate
2-OGDD: 2-Oxoglutarate-Dependent Dioxygenase
ATP: Adenosine Triphosphate
CA: Cinnamaldehyde
CCL1: C-C Motif Chemokine Ligand 1
DCs: Dendritic Cells
ERK1/2: Extracellular Signal-Regulated Kinase 1/2
FLS: Fibroblast-Like Synoviocytes
FOXP3: Forkhead Box P3
G6PD: Glucose-6-Phosphate Dehydrogenase
GABA: Gamma-Aminobutyric Acid
GLUT1: Glucose Transporter 1
GPCR: G protein-coupled receptor
GPR91: G Protein-Coupled Receptor 91
GRK2: G protein-coupled receptor kinase 2
HIF-1α: Hypoxia-Inducible Factor-1α
HK2: Hexokinase 2
IBD: Inflammatory Bowel Disease
IKK: IκB kinase
IL-1β: Interleukin-1β
K48: Lysine 48
LDHA: Lactate Dehydrogenase A
LPS: Lipopolysaccharide
MMPs: Matrix Metalloproteinases
NADPH: Nicotinamide Adenine Dinucleotide Phosphate
NF-κB: Nuclear Factor Kappa B
NLRP3: NOD-like receptor family, pyrin domain containing 3
OGDHC: Oxoglutarate Dehydrogenase Complex
OXPHOS: Oxidative Phosphorylation
PC: Pyruvate Carboxylase
PDH: Pyruvate Dehydrogenase
PFKFB3: 6-phosphofructo-2-kinase/fructose-2,6-biphosphatase 3
PGAM5: phosphoglycerate mutase family member 5
PHD: Prolyl Hydroxylase
PPP: Pentose Phosphate Pathway
Prdm1: PR Domain Containing 1
RA: Rheumatoid Arthritis
RIP3: receptor-interacting protein kinase 3
ROS: Reactive Oxygen Species
SDH: Succinate Dehydrogenase
SDHB: Succinate Dehydrogenase Subunit B
STAT3: Signal Transducer and Activator of Transcription 3
STUB1: STIP1 Homology and U-Box Containing Protein 1
SUCNR1: Succinate Receptor 1
TCA: Tricarboxylic Acid Cycle
TCR: T Cell Receptor
TET: Ten Eleven Translocation
TGF-β1: Transforming Growth Factor-β1
Th1: T Helper 1 cell
Th17: T Helper Cell 17
TLR: Toll-like receptor
TMEM126B: Transmembrane protein 126B
TNF-α: Tumor Necrosis Factor-alpha
Treg: Regulatory T Cell
VEGF: Vascular Endothelial Growth Factor.
